# Short‐term and long‐term effects of cryoballoon ablation versus antiarrhythmic drug therapy as first‐line treatment for paroxysmal atrial fibrillation: A systematic review and meta‐analysis

**DOI:** 10.1002/clc.24092

**Published:** 2023-07-20

**Authors:** Zirui Liu, Zhengkai Yang, Yu Lu, Haocheng Wang, Cao Zou

**Affiliations:** ^1^ Department of Cardiology First Affiliated Hospital of Soochow University Suzhou China

**Keywords:** antiarrhythmic drugs, atrial fibrillation, cryoballoon ablation, first‐line treatment

## Abstract

Cryoballoon ablation (CBA) is an effective treatment for drug‐refractory atrial fibrillation (AF) patients. Whether CBA as a first‐line treatment is superior in the rhythm control of AF than antiarrhythmic drugs (AAD) remains unclear. CBA is superior to AAD as initial therapy for rhythm control of paroxysmal atrial fibrillation (PAF). A comprehensive database search was performed in PubMed, Embase, Cochrane, and Web of Science from inception to March 22, 2023. Treatment efficacy was pooled using risk ratio (RR) and standardized mean difference (SMD) with a 95% confidence interval (CI). This study was registered with Prospero (CRD42023401596). Five randomized‐controlled trials involving 923 patients and an observational study were included in this study. The CBA group had a significantly lower overall recurrence rate than the AAD group (CBA vs. AAD: RR = 0.59, 95% CI = 0.49–0.71, *p* < .05, *I* ^*2*^ = 0). The incidence of persistent AF could be better controlled in the CBA group than in the AAD (CBA vs. AAD: RR = 0.17, 95% CI = 0.06–0.49, *p* < .05, *I* ^*2*^ = 0). CBA could improve the quality of life (QoL) of patients better than AAD (CBA vs. AAD: SMD = 0.40, 95% CI = 0.14–0.67, *p* < .05, *I* ^*2*^ = 68.5%). CBA can reduce hospitalization rate significantly than AAD at 36‐month follow‐up (CBA vs. AAD: RR = 0.29, 95% CI = 0.15‐0.58, *p* < .05, *I* ^*2*^ = 0%). Compared to AAD, CBA as first‐line therapy could reduce the recurrence rate of atrial arrhythmia and incidence of persistent AF and improve QoL in PAF patients with lower incidences of hospitalization.

## Graphical abstract

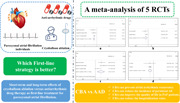

## 1 INTRODUCTION

Atrial fibrillation (AF) is an extremely common rapid atrial arrhythmia that affects approximately 60 million people worldwide.^1^ In most instances, AF starts as a paroxysmal self‐terminating arrhythmia. However, electrical and structural remodeling due to atrial fibrosis can lead to the progression of paroxysmal atrial fibrillation (PAF) to persistent AF in approximately 10% of patients over a 1‐year follow‐up.^2^, ^3^ Persistent AF is not only strongly associated with higher risks of heart failure, cerebral ischemic stroke, and thrombotic events, but it also significantly reduces the quality of life (QoL) of affected patients.^4^, ^5^, ^6^ Therefore, the management and treatment of PAF have gained increasing attention in clinical practice.

Antiarrhythmic drugs (AADs) and catheter ablation (CA) are common treatments for AF.^7^ Recent guidelines recommend that AADs should be the preferred medications for PAF patients and CA should be considered for patients with drug refractory.^7^, ^8^, ^9^ However, AAD therapy is unable to control the rhythm of AF in nearly 70% of patients^10^ and this consequently prolongs the treatment cycle. Previous studies have revealed the importance of the time between the first diagnosis of AF and ablation, also known as the diagnosis‐to‐ablation time (DAT).^11^, ^12^, ^13^ It was found that a shorter DAT (less than 1 year) could lead to a lower recurrence rate and fewer hospitalizations,^11^ which suggested the potential clinical implication of first‐line CA strategy.

Radiofrequency ablation (RFA) and cryoballoon ablation (CBA) are currently the two major CA techniques for PAF treatment and they differ in the energy source.^9^ The famous “FIRE AND ICE” trial showed that CBA was noninferior to pulmonary vein isolation by RFA in efficacy and safety.^14^ In addition, CBA was superior to RFA in direct‐current cardioversions, repeat ablations, all‐cause rehospitalizations, and cardiovascular rehospitalizations in drug‐refractory symptomatic PAF patients.^15^ To date, only a few studies have investigated the role of CBA as an initial treatment for PAF.^14^, ^16^, ^17^, ^18^, ^19^, ^20^, ^21^, ^22^ Several randomized‐controlled trials (RCTs) demonstrated the relationship between early CBA and AF rhythm control.^16^, ^18^ Additionally, CBA as a first‐line strategy has been shown to be associated with improved QoL.^17^, ^19^, ^20^, ^23^, ^24^ However, these studies reported different outcomes when comparing CBA and AAD. Therefore, whether CBA as first‐line treatment has better effects than AAD on AF rhythm control, safety, hospitalization, and QoL of PAF patients remains unclear.

To address these research gaps, we systematically reviewed existing articles to clarify the efficacy of CBA in AF rhythm control, improving QoL, and decreasing the incidences of serious adverse events (SAEs) compared with AAD.

## 2 METHODS

This systematic review and meta‐analysis were conducted in accordance with the Preferred Reporting Items for Systematic Reviews and Meta‐Analysis guidelines.^25^ This study was registered on PROSPERO (CRD42023401596) and there were no major deviations from the published protocol in PROSPERO. All authors declare that all supporting data are available within the article and in the Supporting Information: Data.

### 2.1 Search strategy

Relevant studies were identified by two investigators (Z. L. and Y. L.) independently by systematically searching Embase, PubMed, Cochrane Library, and Web of Science from inception to March 22, 2023, using the terms “atrial fibrillation” and “cryoballoon ablation.”

### 2.2 Eligibility criteria

Included studies met the following criteria:

(1) Study types included RCTs and observational studies;
(2) study participants were adults with PAF, who had a structurally normal heart and had not received daily AAD therapy;
(3) interventions included CBA or AAD therapy with at least 12 months of follow‐up; and
(4) studies reported at least one of the following outcomes: efficacy, safety, recurrence rate, QoL, and/or the incidence of persistent AF.

### 2.3 Exclusion criteria

Excluded studies met the following criteria:

(1) Including participants under the age of 18 or animals;
(2) focusing on treatment for persistent or drug‐refractory AF patients;
(3) comparing the efficacy and safety between CBA and RFA as the initial therapy; and
(4) lacking full texts, such as abstracts from conferences or review articles, or letters to editors.

### 2.4 Data extraction and quality assessment

Data were extracted by two independent investigators (Z. L. and Y. L.), and consistency in the extracted data was verified by a third investigator (H. W.). Any disagreement was resolved through discussion and consensus. The extracted data were verified by the reviewers and duplicates were removed using Endnote X9 (Clarivate Analytics). A structured data collection form was used to record the baseline characteristics of the studies including name, year of publication, study design type, and demographics. Additionally, left atrial diameter, left ventricular ejection fraction, and underlying diseases associated with side effects were also recorded, including hypertension, coronary artery disease, previous stroke, and transient ischemic attack. Various outcomes including the recurrence rate, QoL, SAEs, hospitalization, and intervention‐related side effects were also extracted. The quality of RCTs was assessed using the Cochrane risk‐of‐bias tool for randomized trials version 2 (ROB 2)^26^ by two independent investigators (Z. L. and Z. Y.). The RoB 2 tools have been developed from the original ROB 1,^27^ which specifically caters to trials with either parallel or crossover designs. The tool is structured into a fixed set of domains, which enables the evaluation of various aspects of trial design, conduct, and reporting, such as randomization, blinding, discrepancies in baseline levels, measurement, missing, and selection of outcome data. An algorithm is utilized to calculate the risk of bias, based on responses to the signaling questions, and is expressed as either a “low” or “high” risk of bias or the intermediate option of “some concerns of” risk of bias. Publication bias was assessed using the funnel plot analysis. The asymmetry of the funnel plot was assessed using Egger's regression test.^28^

### 2.5 Study outcomes

#### 2.5.1 Primary outcome

The primary outcome of this meta‐analysis was the rhythm control of PAF, which included the recurrence rate of any symptomatic and asymptomatic atrial tachyarrhythmias at 1‐year follow‐up and the incidence of persistent AF at 3‐year follow‐up.

#### 2.5.2 Secondary outcomes

The secondary outcomes included QoL measured by the Atrial Fibrillation Effect on Quality‐of‐Life (AFEQT) questionnaire, hospitalization, intervention‐related side effects, and SAEs. Atrial arrhythmia recurrence was defined as AF, atrial tachycardia, or atrial flutter for ≥30 seconds during ambulatory monitoring or for ≥10 seconds on a 12‐lead electrocardiogram (ECG).^9^ Persistent AF was defined as instances of AF lasting for more than 7 days during a 7‐day Holter ECG monitoring or necessitating termination through electrical cardioversion after 48 hours.^8^ SAE is defined as an event that results in permanent impairment of a bodily function or structure, serious deterioration in health causing life‐threatening injury or illness, inpatient hospitalization, or prolonged hospitalization for more than 1 day, the requirement of surgical or medical intervention to prevent a life‐threatening illness or injury or to permanently impair a body structure or function, or death.

### 2.6 Statistical analysis

The efficacy of treatment was evaluated using risk ratio (RR), standardized mean difference (SMD), and 95% confidence interval (CI). A fixed effects model was used when heterogeneity was low (*I* ^*2*^ < 50% or *p* ≥ .05)^29^; otherwise, a random effects model was used (*I* ^*2*^ > 50% or *p* < .05). The sensitivity analysis was performed to determine the robustness of the results. Outcomes of interest were analyzed using StataSE 15. A *p* < .05 was considered statistically significant.

## 3 RESULTS

### 3.1 Search and screening results

**Figure 1:**
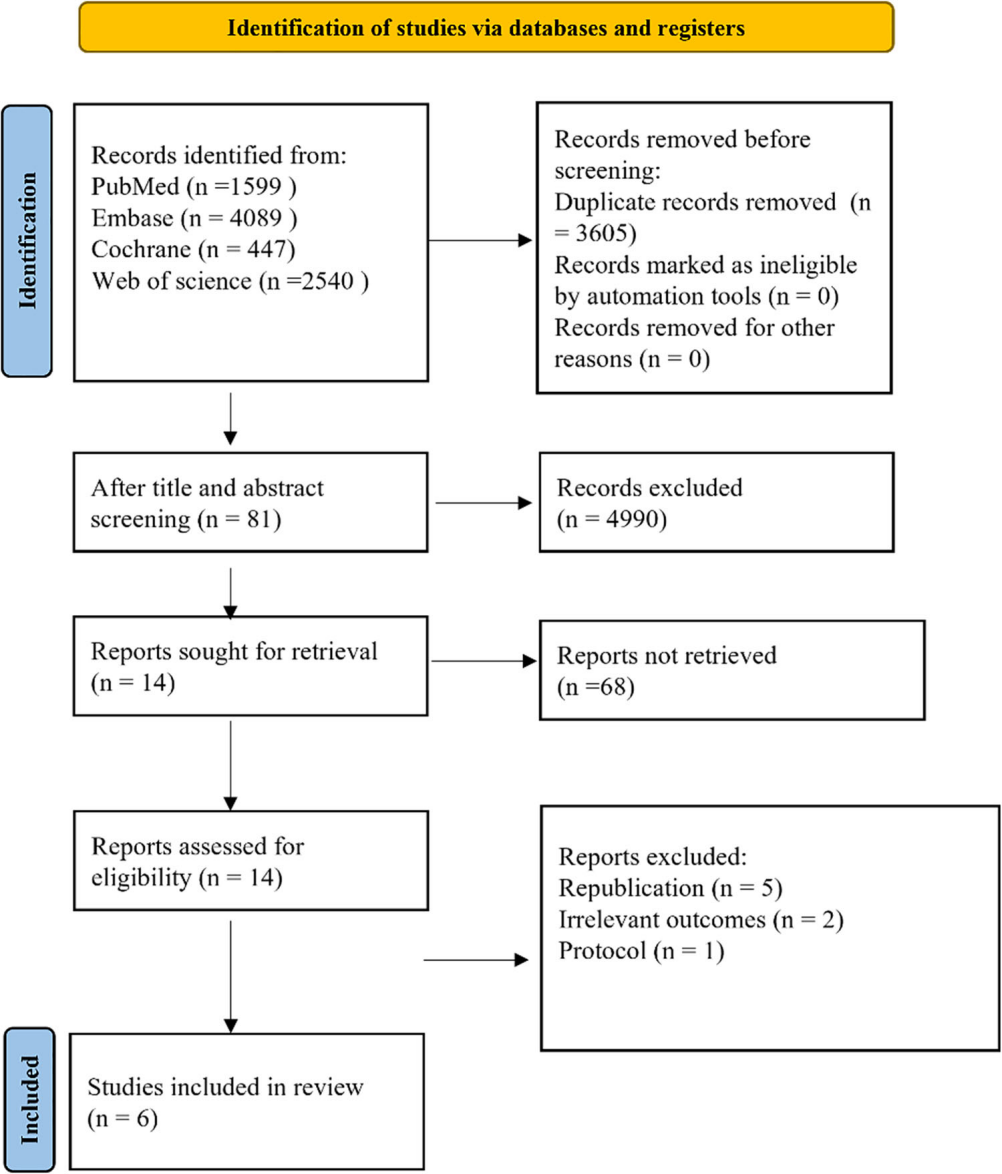
Preferred Reporting Items for Systematic Reviews and Meta‐Analysis diagram of study selection process.

A total of 8675 potential records were retrieved from PubMed (*n* = 1599), Embase (*n* = 4089), Cochrane Library (*n* = 447), and Web of Science (*n* = 2540), and 3605 duplicates were removed. After screening the title and abstract, the full texts of the remaining records were reviewed to select studies that met the eligibility criteria. A final total of 5 eligible RCTs involving 928 participants (467 patients in the first‐line CBA group and 461 patients in the AAD group) were included in this meta‐analysis^16^, ^17^, ^18^, ^19^, ^20^ (Figure 1). An observational study^21^ was also included without data analysis due to the lack of studies.

### 3.2 Study characteristics

The baseline characteristics of the five included RCTs are listed in Supporting Information: Table S1. The five RCTs (EARLY‐AF, STOP‐AF, Cryo‐First, PROGRESSIVE‐AF, and a Chinese study), which were published between 2020 and 2023, were conducted in Canada (*n* = 2), China (*n* = 1), the United States (*n* = 1), and Germany (*n* = 1). There were 467 patients in the CBA group and 461 patients in the AAD group. It is worth noting that PROGRESSIVE‐AF included the same patients as EARLY‐AF, but the former focused on the incidence of persistent AF after intervention, which is in accordance with the Chinese study.

### 3.3 Quality assessment

We used the risk of bias tool RoB 2 (revised version 2019)^26^ to assess the risk of bias in the included RCTs. Using RoB 2, the risk of bias among the RCTs analyzed was estimated (Supporting Information: Figure S1). All RCTs had a low risk or some concerns of bias in most domains of the RoB 2, Supporting Information: Figure S1). However, the absence of blinding in the trial may have contributed to the observed advantages of ablation, including the enhancement in the overall QoL.

### 3.4 Meta‐Analysis

#### 3.4.1 Primary outcomes

**Figure 2:**
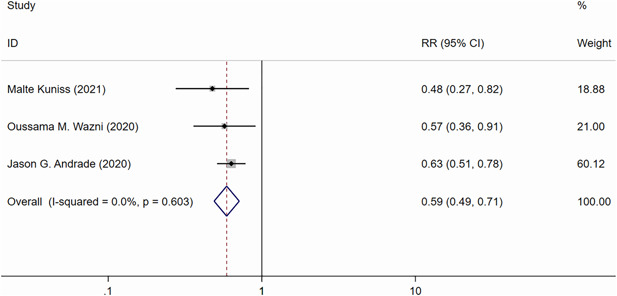
Forest plot of the recurrence of atrial tachyarrhythmias at 1 year. CI, confidence interval; RR, risk ratio.

Three RCTs^17^, ^19^, ^20^ (*n* = 724) reported the recurrence rate of any atrial tachyarrhythmia (symptomatic/asymptomatic AF, atrial flutter, or atrial tachycardia) at the 1‐year follow‐up. The CBA group had a significantly lower overall recurrence rate compared with the AAD group (CBA vs. AAD: RR = 0.59, 95% CI = 0.49–0.71, *p* < .05) (Figure 2). No significant heterogeneity was detected among the studies (*I* ^*2*^ = 0%, *p* = .603).

**Figure 3:**
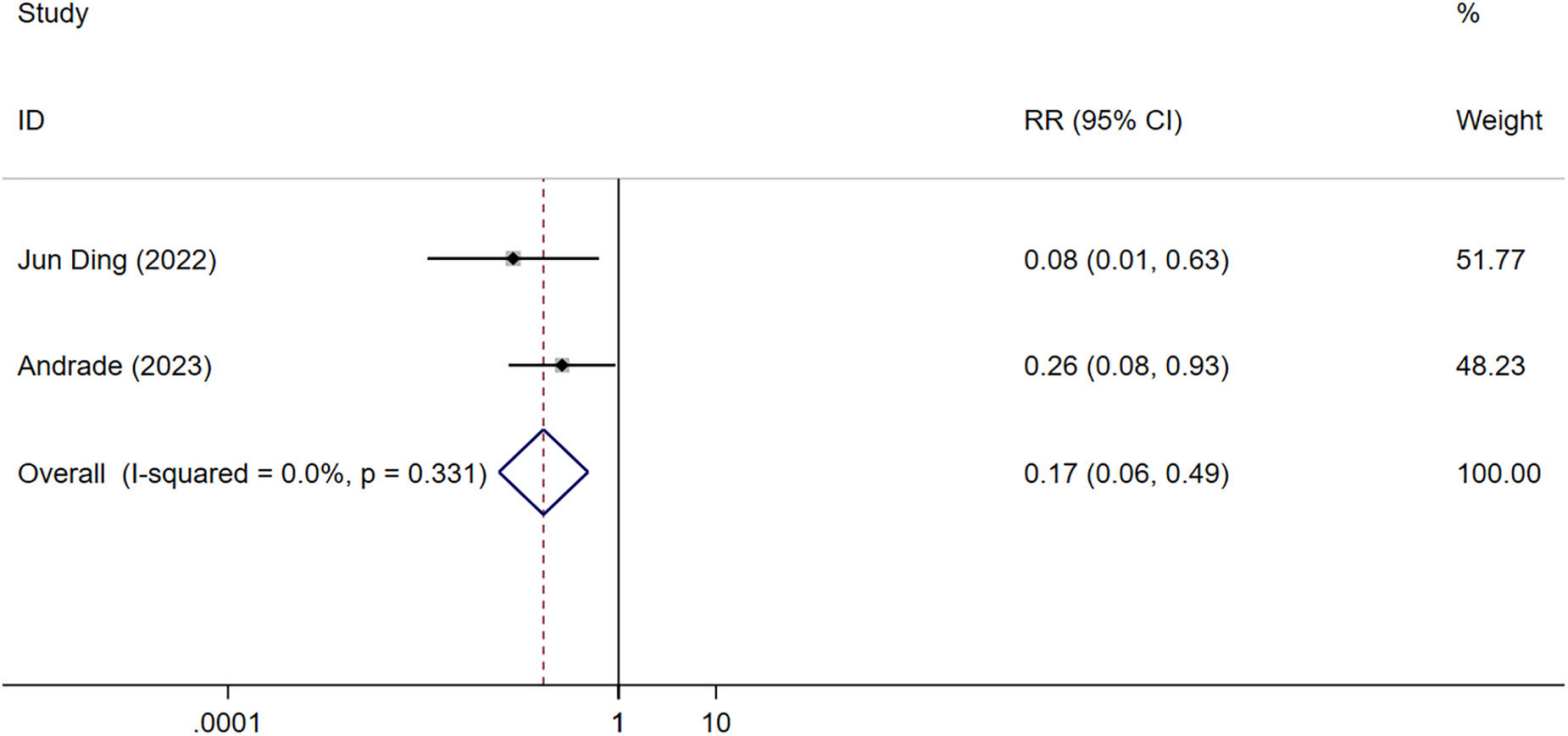
Forest plot of the incidence of persistent atrial fibrillation at 3 years. CI, confidence interval; RR, risk ratio.

Two RCTs^16^, ^18^ (*n* = 507) reported the incidence of persistent AF at 3 years. A significantly lower incidence of persistent AF was observed in the CBA group than in the AAD group (CBA vs. AAD: RR = 0.17, 95% CI = 0.06–0.49, *p* < .05) (Figure 3). No heterogeneity was detected between the studies (*I* ^*2*^ = 0%, *p* = .331).

#### 3.4.2 Secondary outcomes

**Figure 4:**
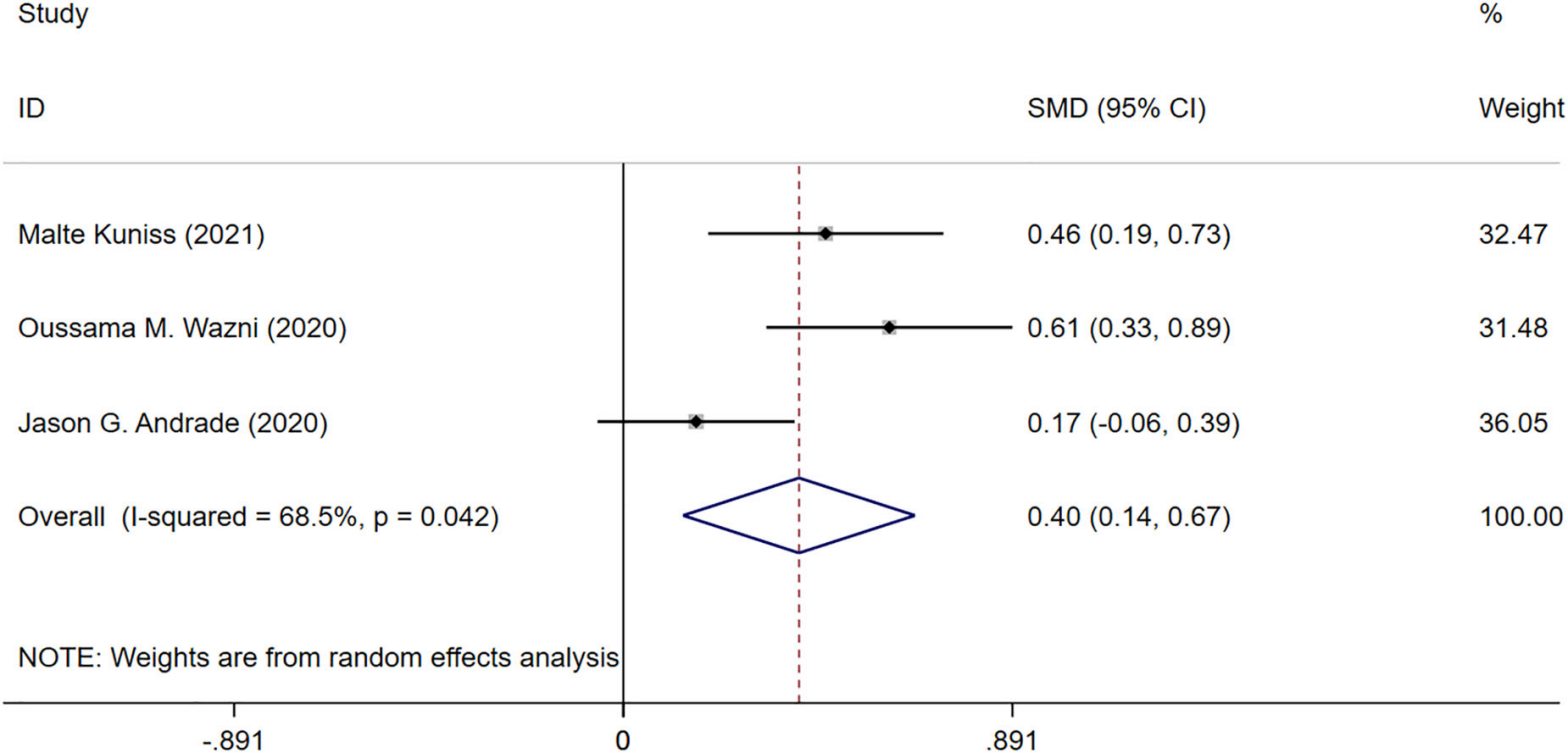
Forest plot of the quality of life of paroxysmal atrial fibrillation patients at 3 years. CI, confidence interval; SMD, standardized mean difference.

Three RCTs (*n* = 724) evaluated the QoL of PAF patients using the AFEQT questionnaire.^17^, ^23^, ^24^ CBA could significantly improve the QoL compared with the AAD groups (CBA vs. AAD: SMD = 0.40, 95% CI = 0.14–0.67, *p* < .05) (Figure 4). However, there was also significant heterogeneity among the studies (*I* ^*2*^ = 68.5%, *p* = .042).

**Figure 5:**
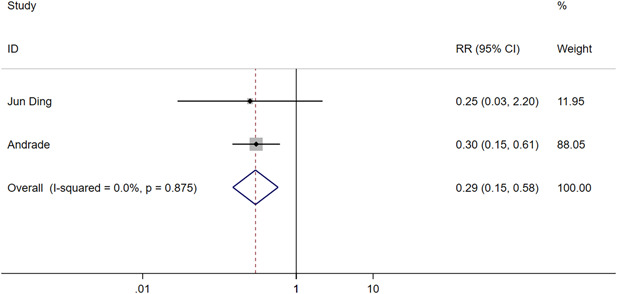
Forest plot of the 3‐year cumulative hospitalization of paroxysmal atrial fibrillation patients. CI, confidence interval; RR, risk ratio.

Two RCTs.^16^, ^18^ reported cumulative hospitalization over 3 years, which suggested that CBA can reduce hospitalization rate significantly than AAD (CBA vs. AAD: RR = 0.29, 95% CI = 0.15–0.58, *p* < .05) (Figure 5).

SAEs were assessed for each patient in all five RCTs.^16^, ^17^, ^18^, ^19^, ^20^ There was no significant difference in the incidence of SAE between the CBA and AAD groups at both the 1‐year follow‐up (CBA vs. AAD: RR = 0.81, 95% CI = 0.57–1.14, *p* < .05)^17^, ^19^, ^20^ and 3‐year follow‐up (CBA vs. AAD: RR = 0.57, 95% CI = 0.30–1.08, *p* < .05)^16^, ^18^ (Supporting Information: Figures S2 and S3). No heterogeneity was detected among the studies.

Other adverse events affecting the heart, lungs, and gastrointestinal tract were also reported in the RCTs. Meta‐analysis showed that the CBA group had a significantly lower incidence of heart adverse events at the 1‐year follow‐up (Supporting Information: Table S2).

### 3.5 Sensitivity analysis and publication bias

A sensitivity analysis of all outcomes was performed, wherein we recalculated the effect sizes in our meta‐analysis by omitting one study at a time. We observed that omitting a single study each time showed no substantial modification of the overall effect, which proved the robustness of the results. These results can be found in Supporting Information: Appendix (Supporting Information: Figures [Link], [Link], [Link], [Link]). However, we did not use a funnel plot or Egger's test to assess publication bias in this meta‐analysis due to the limited number of included studies.

## 4 DISCUSSION

In this systematic review and meta‐analysis of 5 RCTs involving 928 PAF patients, we compared the efficacy of CBA versus AAD in the rhythm control of PAF at the 1‐ and 3‐year follow‐up. We found that CBA significantly reduced the recurrence rate of any atrial arrhythmia and the incidence of persistent AF during the follow‐up period compared with AAD, which indicates that first‐line CBA is an effective method for AF rhythm control. In addition, CBA was superior to AAD in improving the QoL of PAF and was associated with a lower hospitalization rate. Consistent with previous meta‐analysis, there was no significant difference in the incidences of SAEs between the CBA and AAD groups,^30^, ^31^ demonstrating that CBA is a safe treatment for PAF patients.

AF is a progressive disease that can be life‐threatening. Left atrial fibrosis has been shown to play a key role in the pathophysiology of AF,^2^ and the area of fibrosis increases as AF duration increases. The DECAAF trial^32^ showed that atrial fibrosis was independently associated with the possibility of recurrent arrhythmia. For every 1% increase in the left atrial fibrotic area, the unadjusted overall hazard ratio for arrhythmia recurrence was 1.06 (95% CI, 1.03–1.08; *p* < .001). However, the subsequent DECAAF II trial found no significant difference in recurrence rate between the CA and CA plus magnetic resonance imaging‐guided fibrosis ablation groups,^33^ which emphasized the importance of early ablation strategy. Some studies found that AADs can temporarily prevent AF recurrence but failed to reverse atrial structural remodeling,^34^, ^35^ while CBA is strongly associated with substantial reversal of the adverse structural remodeling by means of pulmonary venous isolation. These findings indicate that early AF intervention may be beneficial to AF rhythm control.

To the best of our knowledge, this is the first systematic review and meta‐analysis to comprehensively evaluate the short‐term and long‐term effects of first‐line CBA on PAF rhythm control. It is important because 3%–5% of patients who had successful CA eventually relapsed in a year.^36^ Many of these patients were recommended to take AADs as first‐line treatment,^9^ whereas CA was generally offered after the failure of AADs. Several recent studies have reported that first‐line CBA^22^, ^30^, ^31^, ^37^ can substantially lower AF recurrence within 12 months compared with AAD. However, whether early ablation in PAF can prevent the progression of atrial structural and cellular changes that lead to persistent and permanent AF has been controversial.^38^ The EAST‐AFNET4 trial showed that CBA was beneficial for rhythm control in all patients with recently diagnosed AF.^39^ Meanwhile, the notion of shortening the DAT also suggests early CBA may prevent PAF recurrence. In line with this, a study showed that the rate of 1‐year AF recurrence decreased by 27% after CBA.^12^ However, further RCTs are warranted to confirm this conclusion. Our meta‐analysis of two RCTs^16^, ^18^ affirmed that first‐line CBA can significantly decrease the long‐term incidence of persistent AF, which is consistent with the FREEZE trial^14^ and another observational study included in this meta‐analysis.^21^

Our work also indicated that the incidences of SAEs were similar between the CBA and AAD groups at both the 1‐ and‐3‐year follow‐up, which further confirmed the safety of first‐line CBA. This outcome is also consistent with the FREEZE study,^14^ which compared safety between RFA and CBA in PAF patients. However, additional high‐quality RCTs evaluating the safety and efficacy of CBA in initially diagnosed PAF patients are warranted.

### 4.1 Limitations

There are several limitations in this systematic review and meta‐analysis. First, despite our systematic and comprehensive review, only a few studies were included in our meta‐analysis and hence publication bias could not be assessed. Second, all RCTs compared the efficacy of surgical and pharmaceutical treatments, rendering blinding impractical. This may result in an overestimation of the benefits of ablation. However, all experiments were conducted with concealed allocation to ensure rigorous research quality. Third, using intermittent ECG monitoring or symptoms in Ding's trial may have underestimated the incidence of persistent AF in long‐term follow‐up. Last, the rate of loss to follow‐up is also an inevitable problem that will need to be tackled in subsequent trials. Nonetheless, further high‐quality studies are needed to address these limitations and verify our findings.

## 5 CONCLUSION

Compared to AADs, first‐line CBA can effectively prevent atrial arrhythmia recurrence and lower the incidence of persistent AF. Furthermore, this strategy can improve QoL and reduce hospitalization rates without increasing the incidences of SAEs in PAF patients. Although our research has certain limitations, our findings provide valuable insights into new treatment strategies for PAF.

## AUTHOR CONTRIBUTIONS

**Cao Zou**: Conceptualization. **Zirui Liu**: Methodology, formal analysis, and investigation. **Zirui Liu, Zhengkai Yang, Yu Lu**, and **Haocheng Wang**: Writing—original draft preparation. **Zou Cao** and **Zirui Liu**: Writing review and editing. **Cao Zou**: Resources and supervision. All authors commented on previous versions of the manuscript. All authors read and approved the final manuscript.

## CONFLICT OF INTEREST STATEMENT

The authors declare no conflict of interest.

## Supporting information


**Figure S1**: Risk of bias. (A)Risk of bias domains of included RCTs (traffic light plot). (B) Risk of bias summary.

**Figure S2**: Forest plot of the incidence of SAE at 1 year.

**Figure S3**: Forest plot of the incidence of SAE at 3 years.

**Figure S4**: Sensitive analysis of the recurrence of atrial tachyarrhythmias at 1 year.

**Figure S5**: Sensitive analysis of the incidence of persistent AF at 3 years.

**Figure S6**: Sensitive analysis of the QoL of PAF patients at 3 years.

**Figure S7**: Sensitive analysis of the 3‐year cumulative hospitalization of PAF patients.

Supporting information.

## Data Availability

Data sharing is not applicable to this article as no datasets were generated or analyzed during the current study.
