# Supplementary figures and images for: Short‐term and long‐term effects of cryoballoon ablation versus antiarrhythmic drug therapy as first‐line treatment for paroxysmal atrial fibrillation: A systematic review and meta‐analysis
Source: Clin Cardiol. 2023 Jul 20;46(10):1146–53. doi: 10.1002/clc.24092 (PMC10577536; doi:10.1002/clc.24092)

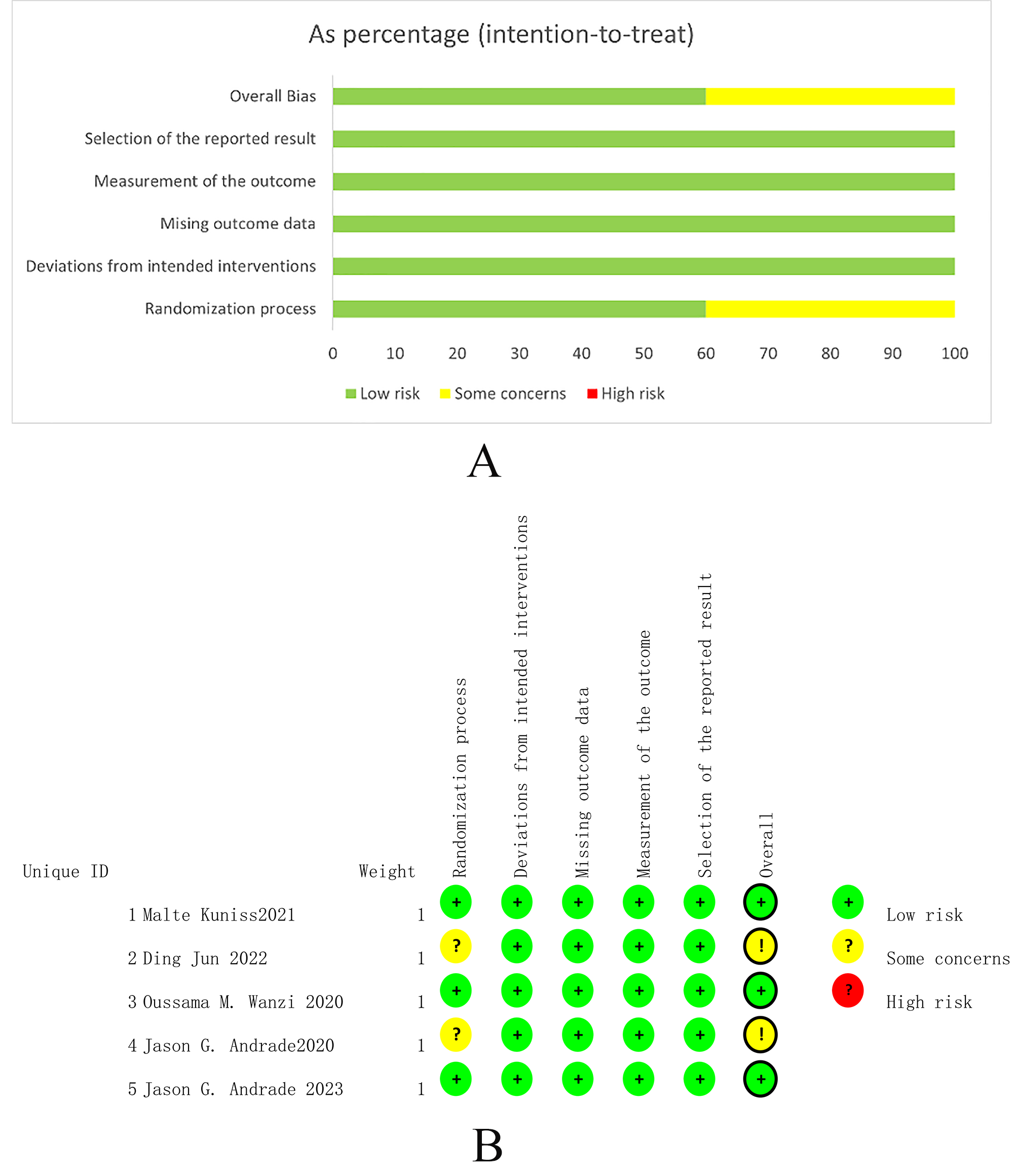

Supplement: Supplementary file 1 — Figure S1: Risk of bias. (A)Risk of bias domains of included RCTs (traffic light plot). (B) Risk of bias summary. [file CLC-46-1146-s002.tif]

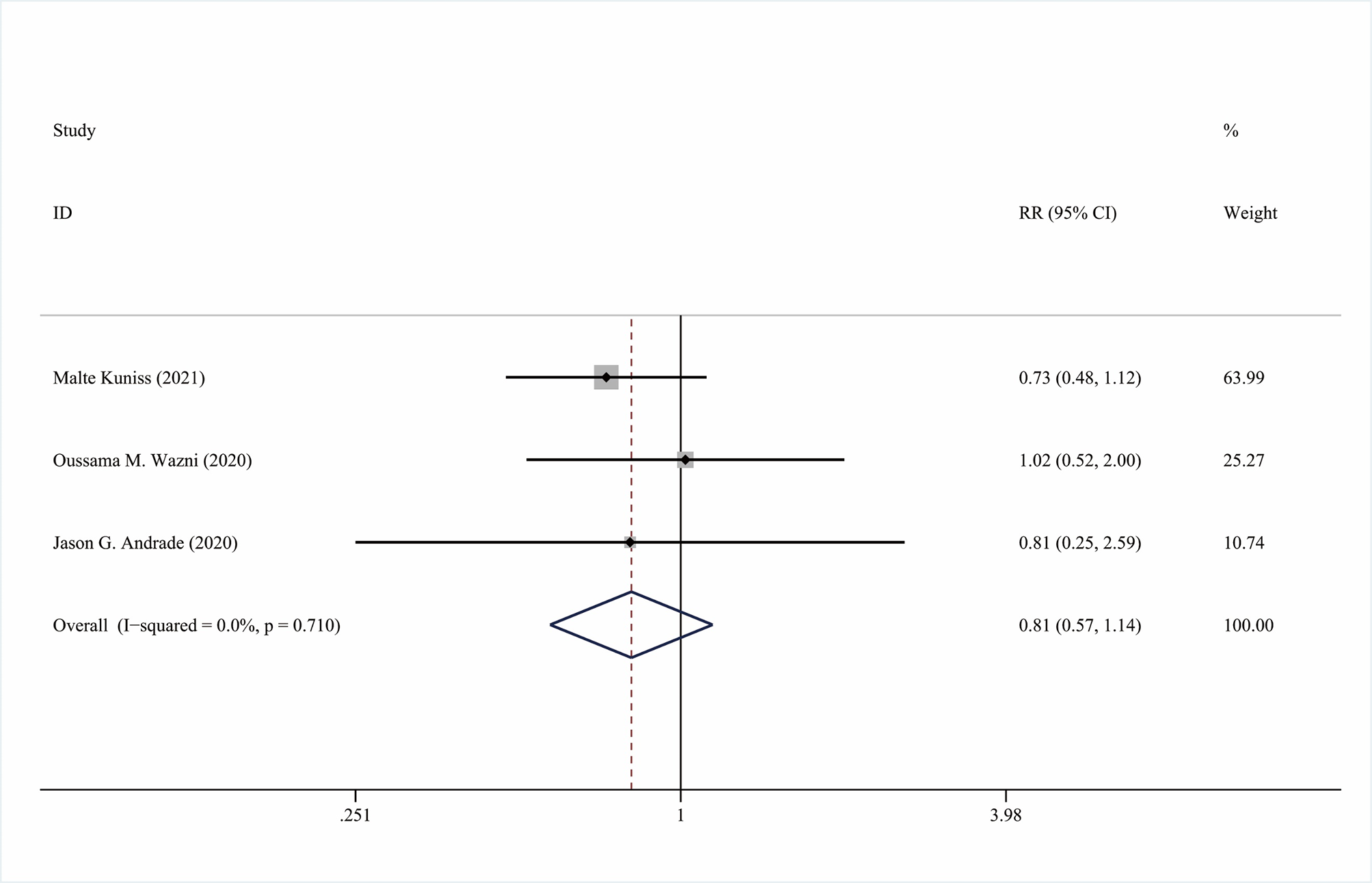

Supplement: Supplementary file 2 — Figure S2: Forest plot of the incidence of SAE at 1 year. [file CLC-46-1146-s005.tif]

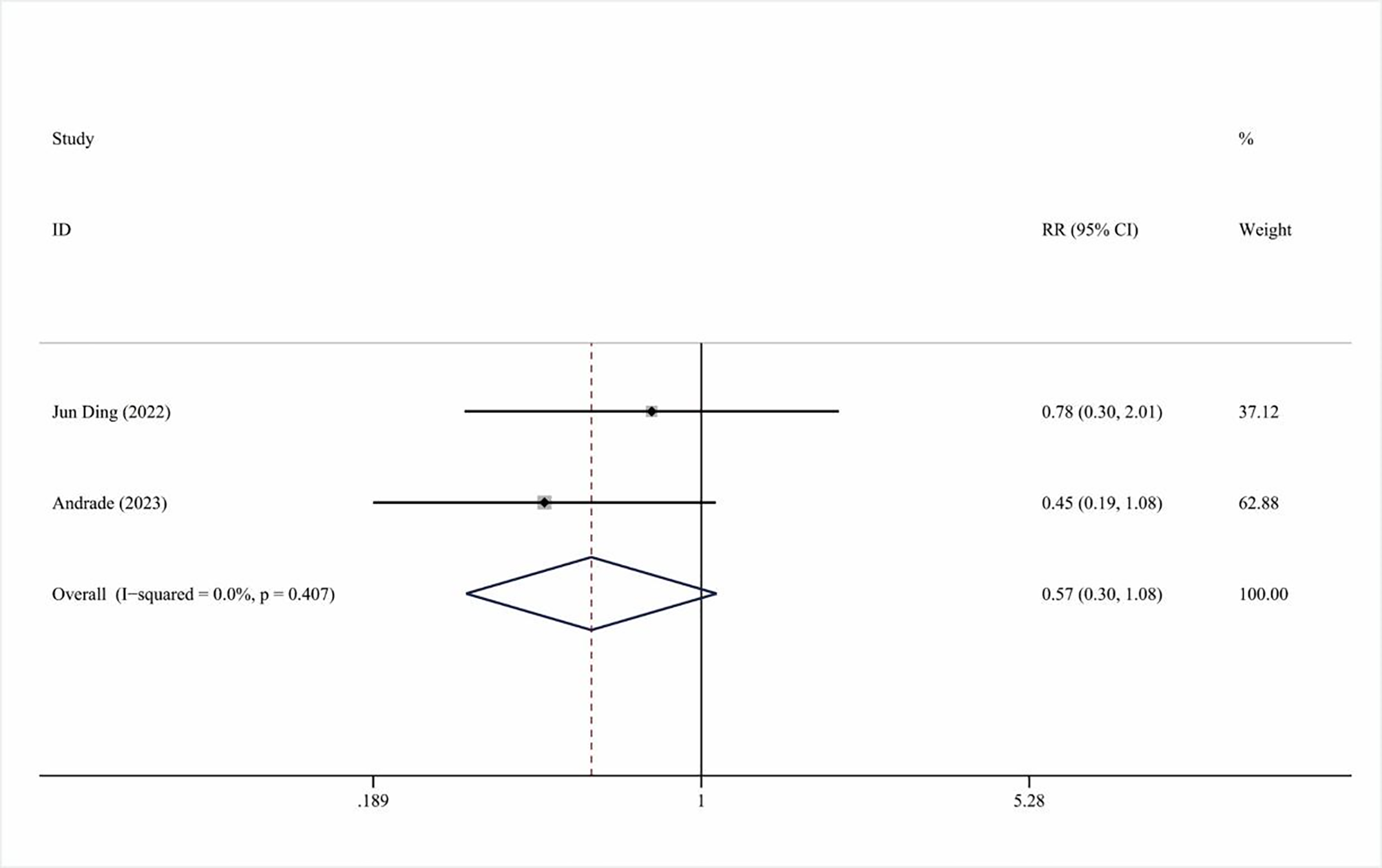

Supplement: Supplementary file 3 — Figure S3: Forest plot of the incidence of SAE at 3 years. [file CLC-46-1146-s007.tif]

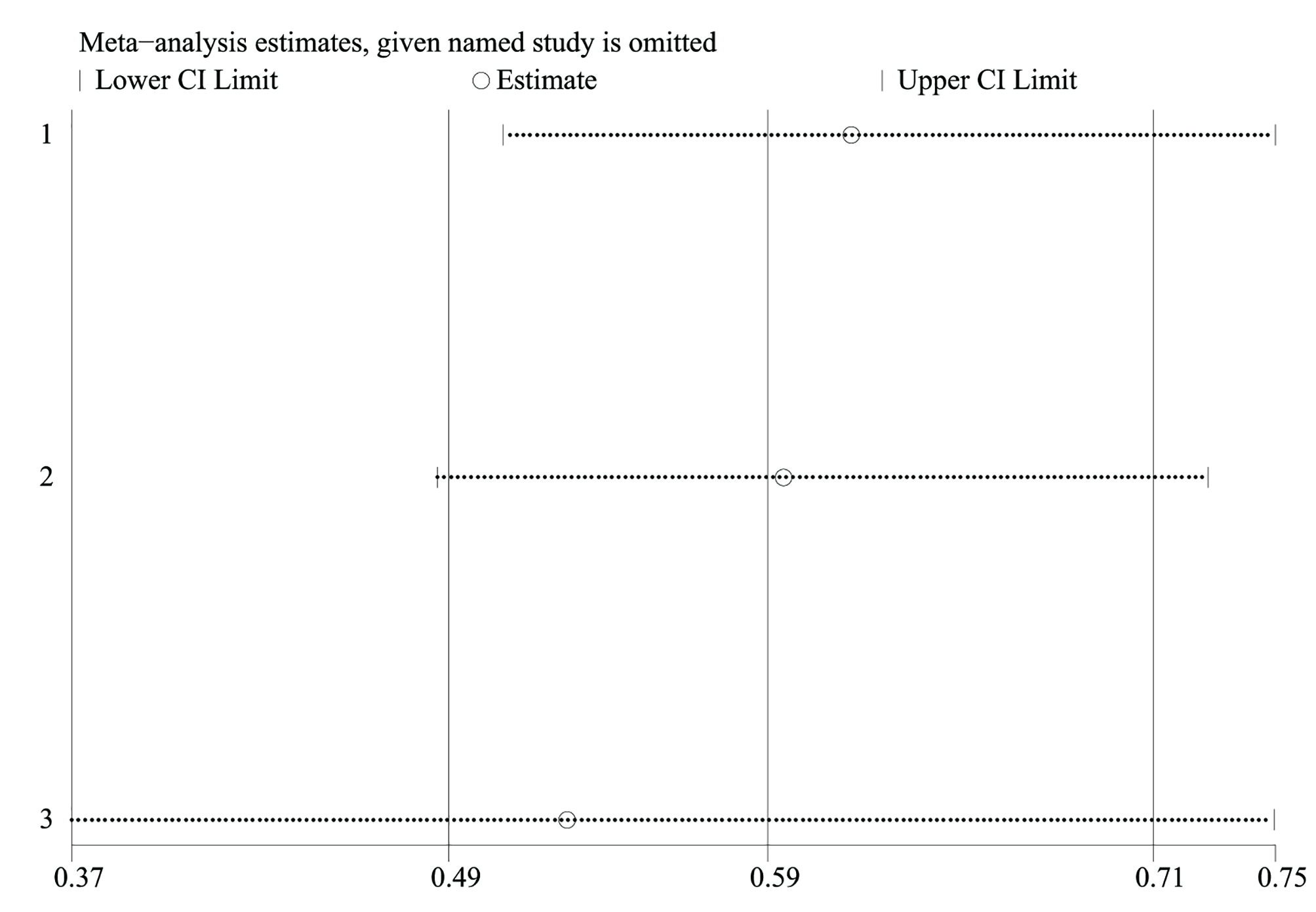

Supplement: Supplementary file 4 — Figure S4: Sensitive analysis of the recurrence of atrial tachyarrhythmias at 1 year. [file CLC-46-1146-s001.tif]

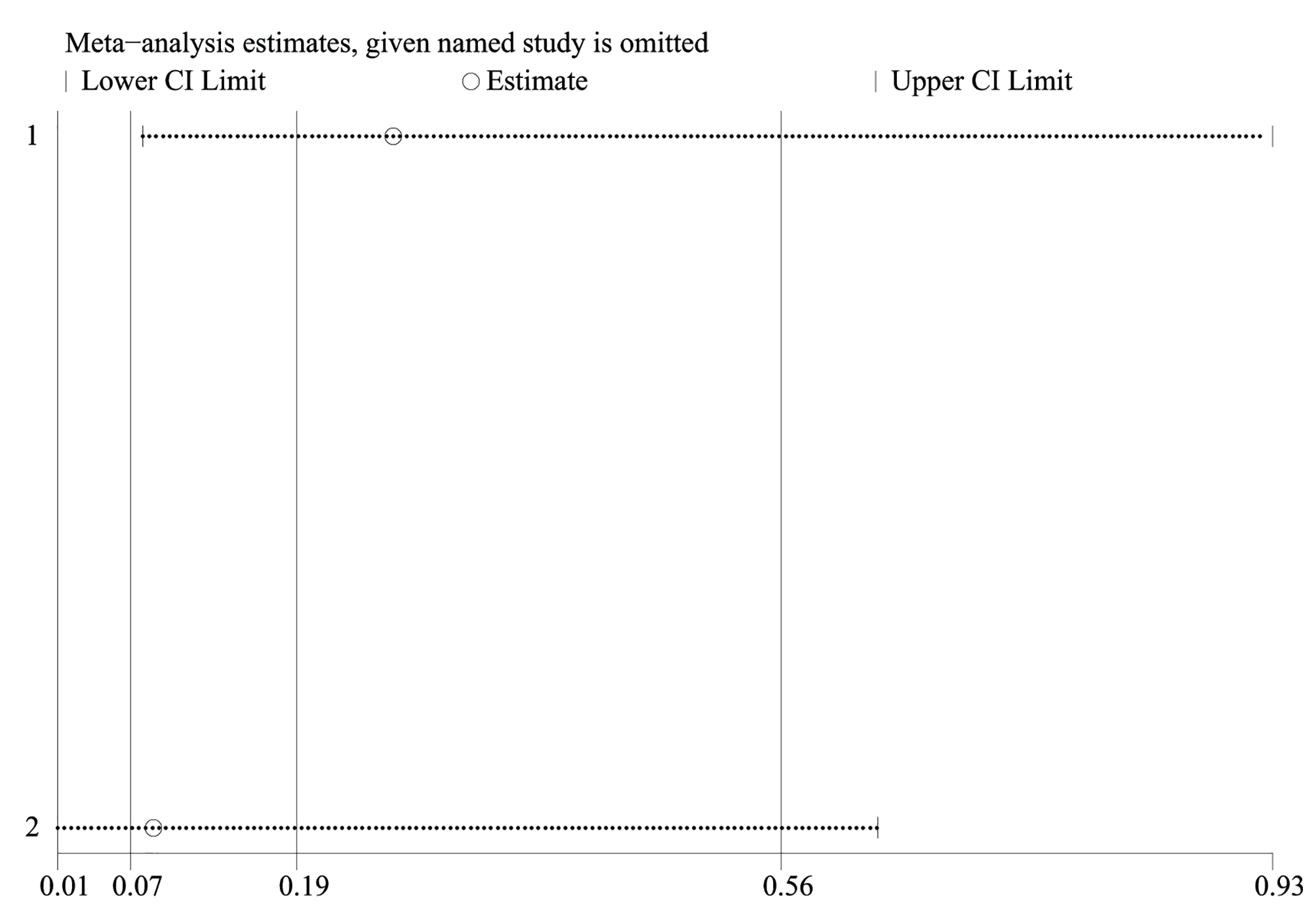

Supplement: Supplementary file 5 — Figure S5: Sensitive analysis of the incidence of persistent AF at 3 years. [file CLC-46-1146-s008.tif]

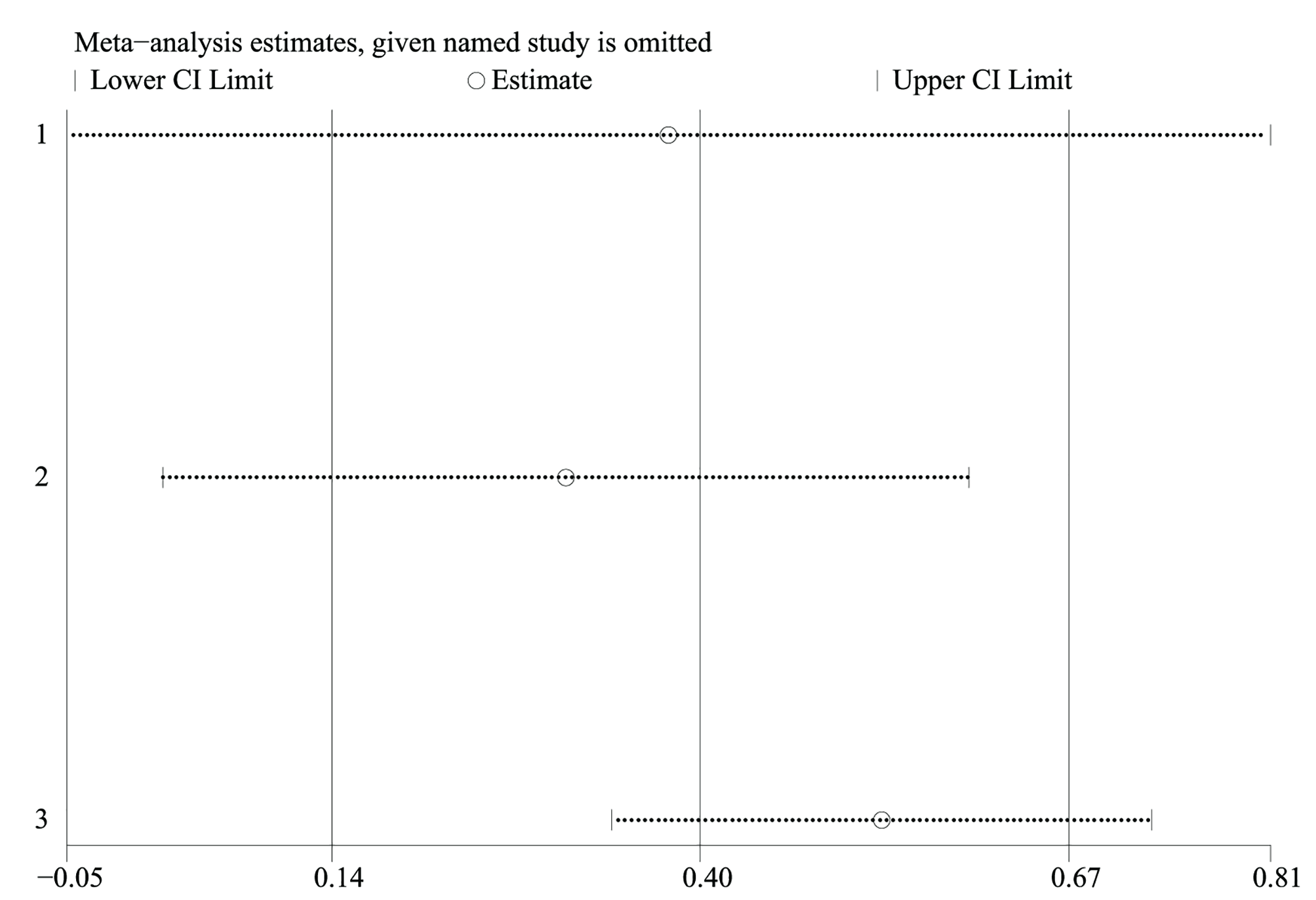

Supplement: Supplementary file 6 — Figure S6: Sensitive analysis of the QoL of PAF patients at 3 years. [file CLC-46-1146-s004.tif]

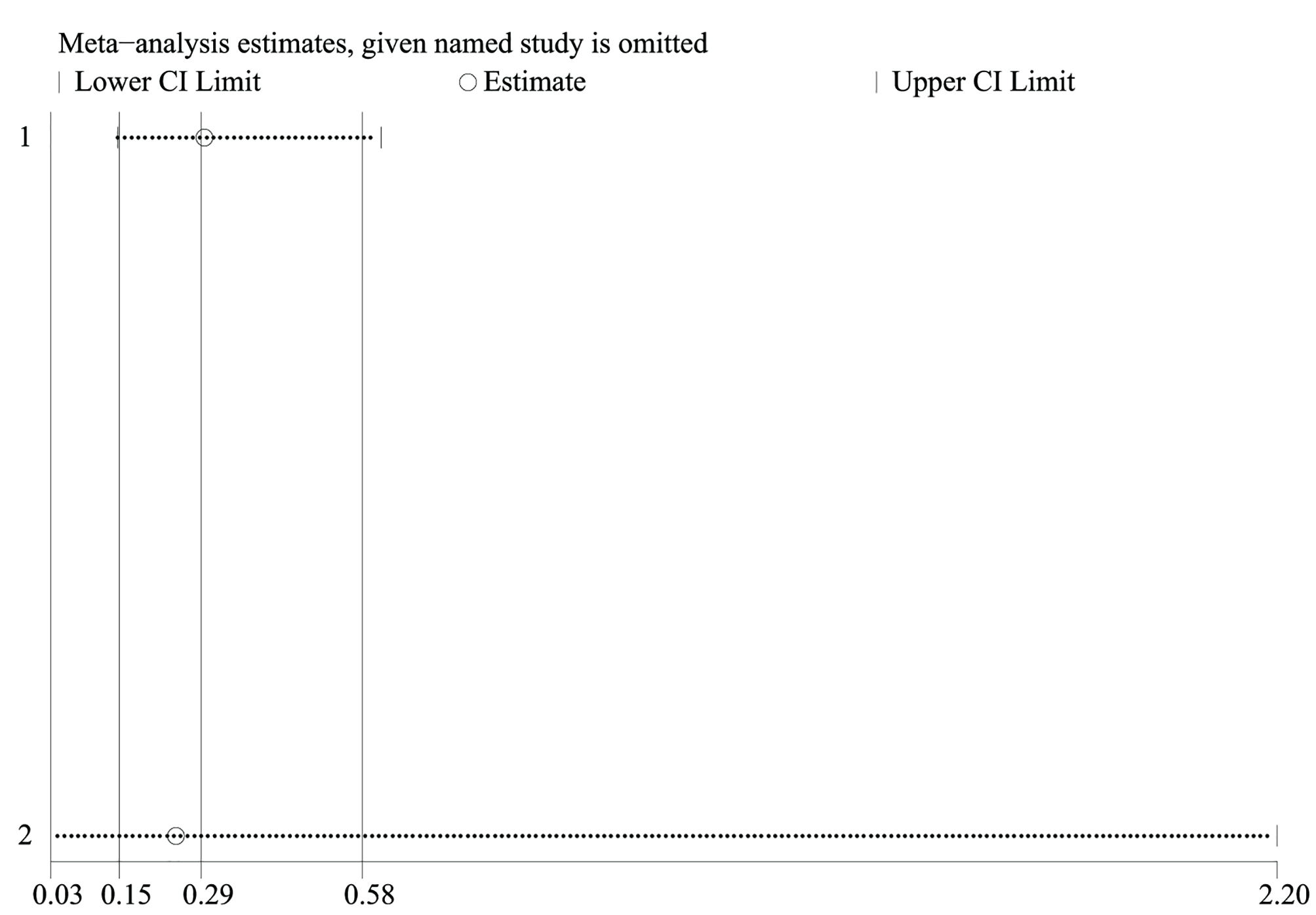

Supplement: Supplementary file 7 — Figure S7: Sensitive analysis of the 3‐year cumulative hospitalization of PAF patients. [file CLC-46-1146-s003.tif]
