# Supplementary material for: Short‐term and long‐term effects of cryoballoon ablation versus antiarrhythmic drug therapy as first‐line treatment for paroxysmal atrial fibrillation: A systematic review and meta‐analysis
Source: Clin Cardiol. 2023 Jul 20;46(10):1146–53. doi: 10.1002/clc.24092 (PMC10577536; doi:10.1002/clc.24092)
Supplement: Supplementary file 8 — Supporting information. [file CLC-46-1146-s006.docx]

**Table S1**: General characteristic of RCTs

| **No.** | 1 | 2 | 3 | 4 | 5 |
| --- | --- | --- | --- | --- | --- |
| **Study(author/year)** | PROGRESSIVE-AF Andrade2023 | Cryo-FIRST  Malte Kuniss2021 | Jun Ding2022 | STOP AF First Oussama M. Wazni2020 | EARLY-AF Jason G. Andrade2020 |
| **IF** | 176.079 | 5.486 | 5.846 | 176.079 | 176.079 |
| **Country** | CAN | Germany | CHN | US | CAN |
| **Design** | RCT | RCT | RCT | RCT | RCT |
| **Muticenter** | Yes | Yes | NO | Yes | Yes |
| **AF type** | PAF | PAF | PAF | PAF | PAF |
| **Follow-up (months)** | 36 | 12 | 36 | 12 | 12 |
| **Patients** | 154/149 | 107/111 | 102/102 | 104/99 | 154/149 |
| **Age** | 57.7±12.3/59.5±10.6 | 50.5±13.1/54.1±13.4 | 60.9±7.89/60.74±10.16 | 60.4±11.2/61.6±11.2 | 57.7±12.3/59.5±10.10 |
| **Male** | 112/102 | 76/72 | 61/60 | 63/57 | 112/102 |
| **LAD** | 39.5±5.0/38.1±6.5 | 37.0±5.9/38.0±4.9 | 38.29±3.68/39.11±3.89 | 38.7±5.7/38.2±5.4 | 39.5±5.0/38.1±6.5 |
| **LVEF** | 59.6±7.0/59.8±7.6 | 62.8±5.4/63.7±5.4 | 60.91±4.71/59.96±5 | 60.9±6.0/61.1±5.9 | 59.6±7.0/59.8±7.6 |
| **Hypertension** | 57/55 | 33/40 | 54/47 | 58/57 | 57/55 |
| **CAD** | 12/7 | 4/1 | 25/27 | 17/14 | 12/7 |
| **prior stroke or TIA** | 4/5 | 0/1 | 8/10 | 2/3 | 4/5 |
| **CBA equipment** | 23-mm or 28-mm  cryoballoon (Arctic Front Advance, Medtronic) | a 28- or 23-mm second-generation cryoballoon (Arctic Front Advance Cardiac Cryoablation Catheter, Medtronic) | 28-or 23-mm second-generation cryoballoon (Arctic Front Advance Cardiac Cryoablation Catheter, Medtronic) | A second-generation cryoballoon  (Arctic Front Advance Cardiac Cryoablation  Catheter, Medtronic) | 23-mm or 28-mm  cryoballoon (Arctic Front Advance, Medtronic) |
| **AAD** | Flecainide  Propafenone  Sotalol Dronedarone  Amiodarone | Flecainide  Propafenone  Sotalol Dronedarone  Amiodarone | Propafenone  Sotalol Dronedarone  Amiodarone | Flecainide  Propafenone  Sotalol Dronedarone  Amiodarone | Flecainide  Propafenone  Sotalol Dronedarone  Amiodarone |

**Table S2:** Adverse events at the 1- and 3-year follow-up

|  | 1-year-follow-up（n=724） | | | | | 3-years-follow-up（n=507） | | | | |
| --- | --- | --- | --- | --- | --- | --- | --- | --- | --- | --- |
|  | CBA（n=365) | AAD（n=359） | *P*-value | RR (95% CI) | *I*^2^% | CBA（n=256） | AAD（n=251） | *P*-value | RR (95% CI) | *I*^2^% |
| Heart | 26 | 47 | 0.01 | 0.56（0.36,0.87） | 25.2 | 9 | 21 | 0.336 | 0.45（0.09,2.29） | 74.7 |
| Pulmonary | 5 | 2 | 0.331 | 1.98（0.50,7.76） | 0.5 | 3 | 1 | 0.394 | 2.29（0.34,15.34） | 0 |
| Stroke/TIA | 1 | 5 | 0.167 | 0.33（0.07,1.60） | 0 | 1 | 4 | 0.0233 | 0.33（0.05,2.05） | 0 |
| Gastrointestinal | 4 | 2 | 0.418 | 2.0（0.77,10.84） | 0 | 4 | 3 | 0.749 | 1.25（0.31,5.00） | 0 |
| Blood vessel | 3 | 0 | 0.215 | 3.99（0.45,36.52） | 0 | 5 | 0 | 0.099 | 5.91（0.72,48.65） | 0 |
